# Impact of the Pre-Dehydration and Drying Methods on the Mass Transfer and Quality Attributes of Yak Milk Casein

**DOI:** 10.3390/foods13071062

**Published:** 2024-03-29

**Authors:** Dong Wang, Zhi Cao, Yumei Gao, Lin Yang, Lili Zhao

**Affiliations:** 1 College of Mechanical & Electrical Engineering, Shaanxi University of Science & Technology, Xi’an 710021, China; wangdong@sust.edu.cn; 2 College of Food Science and Engineering, Northwest A&F University, Yangling 712100, China; mjy19981214@163.com (Z.C.); yumeig2022@163.com (Y.G.); 3 Food Science College, Tibet Agriculture & Animal Husbandry University, Nyingchi 860000, China; yanglin@xza.edu.cn

**Keywords:** pre-dehydration, drying kinetics, drying characteristics, mathematical model, casein

## Abstract

Drying is an important preservation method of casein. Traditional natural draining and drying processes have low efficiency, long processing time, and poor product quality, which urgently need to be improved. This study investigated the effects of pre-dehydration intensities (30 N 30 min (PreD1) and 50 N 30 min (PreD2)) and drying methods (including pulsed vacuum drying (PVD), infrared drying (IRD), and hot air drying (HAD)) on the drying kinetics, drying modeling, and quality of yak milk casein. These findings reveal that PreD2 and PVD both had a positive impact on shortening the drying time. Compared to other combined treatments, PreD2-PVD had the shortest drying time of 6 h. The Midilli–Kucuk mathematical model effectively predicted the drying of casein. The yak milk casein powder treated with PreD2-PVD possessed a higher content of gross compositions, superior color, lower levels of fat oxidation and 5-hydroxymethylfurfural (5-HMF), and higher emulsifying activity index (EAI) and emulsion stability index (ESI) values. Overall, combining pre-dehydration with PVD proved effective in improving the drying rate and maintaining a good quality of yak milk casein, showing promising potential for industrial applications.

## 1. Introduction

Casein is one of the most abundant proteins in dairy products, accounting for about 80% of total milk protein. It is a highly nutritious protein that contains a large amount of essential amino acids, particularly leucine and tryptophan, which are critical for normal physiological functions in humans [1,2]. Yak milk casein (also known as Qula in Tibetan), is primarily produced in the provinces of Gansu, Qinghai, Inner Mongolia, and Xinjiang in China. It is renowned globally for its exceptional quality and functional characteristics. Yak milk casein is widely used in the food, pharmaceutical, cosmetic, coating, and leather industries [3,4,5,6]. However, the majority of these products are low-end and lack added value due to the poor quality of casein [7,8]. Qula is usually made from fresh yak milk by acid precipitation (pH 4.6), drying, dehydration, and granulation [7,9]. That is to say, raw yak milk or skimmed milk is fermented by inoculating starter cultures under natural conditions, leading to the coagulation and subsequent drying of casein. Among them, the dehydration process is a critical unit operation in casein production that directly affects product quality (color, lipid oxidation, and flavor), functional features (solubility and emulsification stability), and microbial contamination [8,10]. During long-term dehydration processing, protein and fat in casein are prone to oxidation and Maillard reactions, resulting in nutrient loss, yellowing, and off-odors. Furthermore, the initial moisture content of yak milk casein is critical to the drying [2]. As casein aggregates, it exhibits strong water-holding capacity. A high moisture content hinders the dispersion and granulation of casein, leading to slower removal of moisture during drying. Moreover, the drying can lead to the formation of a hardened crust on the surface of the aggregates, impeding the diffusion of internal moisture and prolonging drying time [11]. Hence, physical pre-dehydration in combination with drying is a critical step in the drying of casein.

The traditional sun drying methods for casein typically involve natural drainage, leaving approximately a 60–70% residual moisture content [8]. The process of drying typically necessitates a duration of 2 to 3 days, which can lead to spoilage and the production of suboptimal coloration, solubility, and emulsification stability, as well as undesirable flavor and fat oxidation [3,8]. Thus, emerging approaches that rapidly remove most of the moisture from the fermentation coagulum, shorten drying times, and minimize occurrences of Maillard reactions, lipid oxidation, and microbial growth during drying are crucial for enhancing casein quality. Perforated sleeve extrusion dewatering as an alternative pre-dehydration technology has been explored to remove moisture quickly from casein according to the strength of extrusion. After that, pulsed vacuum drying (PVD) was employed to remove the residual water content. It has been found that PVD is beneficial in terms of protecting heat-sensitive compounds, preventing fat oxidation, preserving color, and improving the drying rate [12,13,14]. Infrared drying (IRD) and hot air drying (HAD) are the predominant methods employed in contemporary food drying practices. Consequently, a comparative study was also conducted on these two techniques to explore their efficacy in the drying process [15]. Therefore, in this study, double-mesh sleeve extrusion dehydration combined with drying (PVD, IRD, and HAD) was used to study the effects of the pre-dehydration intensity and drying method on the drying rate, color, solubility, fat oxidation, and other qualities of casein. The technology of pre-dehydration and drying may be interesting for small-scale rural household casein production.

## 2. Materials and Methods

### 2.1. Preparation and Drying of Yak Milk Casein

**Figure 1:**
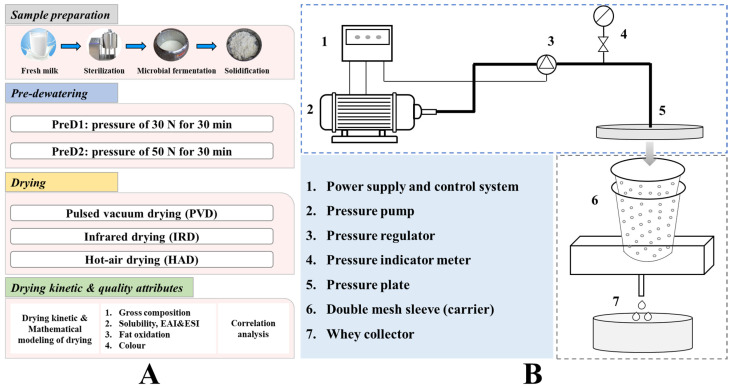
The experimental design (**A**) and schematic diagram of the perforated sleeve extrusion dewatering system (**B**).

**Figure 2:**
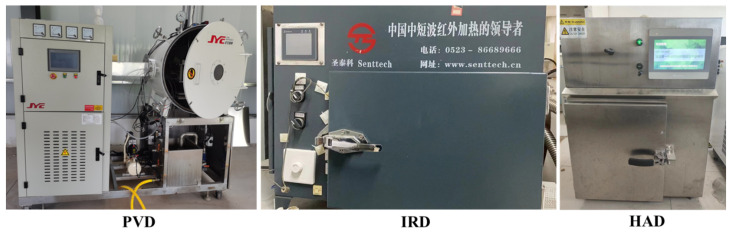
Drying equipment. PVD represents pulsed vacuum drying, IRD represents infrared drying, and HAD represents hot-air drying.

Yak milk-derived casein is renowned globally for its exceptional quality and functional characteristics [7]. Yak milk was collected from 20 healthy yaks in summer, approximately 1–2 months postpartum, from farms located in the Gannan Tibetan Autonomous Region of Gansu Province, China. The collected yak milk was promptly tanked at 4 °C and transported in ice boxes to the laboratory. The fresh yak milk collected was subjected to remove the hair and impurities using an 80-mesh filter, heated at 65 °C for 30 min, and subsequently cooled to 36 ± 2 °C. Based on the preliminary experiments, 0.3% (*w*/*w*) direct starter culture was inoculated, and the mixture was incubated and fermented until a pH of 4.6 was reached [8]. The starter cultures of *Lactobacillus delbrueckii* subsp. *Bulgaricus* (MGD1-3), *Streptococcus thermophilus* (MGB39-5), and *Lactobacillus plantarum* (BM5152) were 2.56%, 1.64%, and 2.45%, respectively. The strains employed in this study were supplied by the Key Laboratory of Dairy Biotechnology and Engineering, Ministry of Education, Inner Mongolia Agricultural University. At this stage, the casein was acidified and coagulated, after which a sample was extracted from the natural drainage process and allowed to drip naturally until no further moisture was present. (The moisture content was measured at this point.) The sample (5 kg) was placed between two meshed barrels with different diameters for pre-dehydration (Figure 1A). The pressure was set at 30 N and 50 N, respectively, and the extrusion process was carried out for 30 min. The pre-dehydration system of casein, illustrated in Figure 1B, mainly consists of a pressure pump, control system, double-layer mesh sleeve with a 5-mesh screen, and liquid collection system. During the extrusion process, the solidified casein is retained in the mesh drum, while the whey liquid passes through the mesh and collects in a liquid container. After being extruded under different pressures, the moisture content of each sample was measured using the method of Cui et al. [1]. Briefly, the gravimetric determination was conducted by drying the samples in a forced-convection oven (DHG-9013A, Shanghai Shanzhi instrument Equipment Co., Ltd., Shanghai, China) at 105 °C until a constant weight was achieved. In addition, the coagulated casein particles, which were of uniform size (with a 10-mesh screen), were manually dispersed and placed on a drying tray with a thickness of 10 ± 2 mm. Subsequently, they were placed in PVD (self-manufactured at the College of Engineering at China Agricultural University, Beijing, China), IRD (Taizhou Senttech Infrared Technology Co., Ltd., Taizhou, China), and HAD (Jiangsu Keheng Environmental Science and Technology Co., Ltd., Zhenjiang, China) chambers for drying (Figure 2). Based on the preliminary experiments, the drying temperature for all three drying methods was set to 40 °C [8]. The pulsed ratio of 12 min: 3 min was employed for PVD, and the convection hot air velocity for both IRD and HAD was set at 2 ± 0.5 m/s [12]. The weight of the samples was collected every hour during drying until the weight difference between two consecutive measurements was less than 0.01 g, indicating that the sample had reached a constant weight.

### 2.2. Moisture Ratio

The moisture ratio (*MR*) of samples was calculated using Equation (1):

(1) $$MR=\frac{M_{t}-M_{e}}{M_{0}-M_{e}}$$

where *M*_t_ = moisture content at t time of drying (g water/g dry matter); *M*_0_ = moisture content of pre-dewatering (g water/g dry matter); and *M*_e_ = equilibrium moisture content (g water/g dry matter).

### 2.3. Mathematical Modeling of Drying

Eight commonly used mathematical models were utilized to describe the moisture changes in samples during one-layer drying (Table 1) [16]. These models are typically developed by applying Fick’s second law, which is a linear equation.

The correlation coefficient (*R*^2^), residual sum of squares (*RSS*), and reduced mean square of the deviation (*χ*^2^) were used to evaluate the goodness of fit, calculated using Equations (2)–(4). The model with the highest *R*^2^ and the lowest values of *RSS* and *χ*^2^ had the best fit [16,21].

(2) $$R^{2}=\frac{\sum_{i=1}^{N}{\left({MR}_{\mathrm{pre},i}\;-\;{MR}_{\exp,i}\right)}^{2}}{\sum_{i=1}^{N}{\left(\bar{{MR}_{\mathrm{pre}}}-{MR}_{\exp,i}\right)}^{2}}$$

(3) $$\mathrm{RSS}=\sum_{i=1}^{N}{\left({MR}_{\mathrm{pre},i}\;-\;{MR}_{\exp,i}\right)}^{2}$$

(4) $$\chi^{2}=\frac{\sum_{i=1}^{N}{\left({MR}_{\exp,i}\;-\;{MR}_{\mathrm{pre},i}\right)}^{2}}{N-z}$$

Here, *MR_exp_*_,*i*_ and *MR_pre_*_,*i*_ represent the moisture ratios measured and predicted by the model, respectively. *N* is the total number of moisture measurements.

### 2.4. Determination of Gross Composition

The Kjeldahl method was used to determine the protein content according to AOAC International (1997) [22] standards, and a conversion factor of 6.38 was used. The fat percentage was determined using the method described by Liu et al. [2]. Briefly, 1.5 mL ammonia, 5 mL ethanol, 10 mL ether, and 10 mL petroleum ether were added to 5.0 g of the sample using a separatory funnel. The samples were fully shaken for 2 min and left at room temperature for 30 min, and the upper layer was collected. The extraction was repeated twice using 5 mL each of ether and petroleum ether. The upper layer was collected, evaporated, and weighed. The ash content was determined by incineration at 530 °C according to the method reported by Cui et al. [1]. Lactose content was determined by subtracting other solid components from casein, as described by Cui et al. [1]. As a control sample, the gross composition of freeze-dried raw milk was also determined. All measurements were performed in triplicate.

### 2.5. Determination of Color

In this study, the CIE LAB color parameters in terms of L*, a*, and b* were utilized to quantitatively describe the surface color changes of samples subjected to different drying methods. The L*, a*, and b* parameters correspond to the lightness spectrum with a range of 0 (black) to 100 (white), red–green spectrum with a range of +60 (red) to −60 (green), and yellow–blue spectrum with a range of +60 (yellow) to −60 (blue), respectively [21]. A colorimeter (CM-5, Hunter Lab, USA) was employed to measure the color parameters of the samples according to the method reported by Wang et al. [21]. In brief, the samples were ground using a low-temperature (GJ-4×100) crusher, placed in Petri dishes (diameter = 5 cm and height = 1 cm), and filled to the top. The colorimeter was calibrated by positioning the measuring head tip flatly onto the surface of a white calibration plate. Following calibration, the L*, a*, and b* values were obtained by measuring the surface of the dried samples. All the analyses were performed in three technical replicates.

### 2.6. 5-Hydroxymethylfurfural

The 5-hydroxymethylfurfural (5-HMF) content of casein was determined by the method of Wang and Wang et al. [8] with slight modifications. In brief, 2.0 g of the sample was treated with 10 mL of 0.15 M oxalic acid, followed by the addition of potassium hexacyanoferrate (3 mL, 90 g/L) and zinc acetate (3 mL, 183 g/L) solutions. After shaking, acetonitrile was added to bring the total volume to 50 mL, and the mixture was centrifuged before filtering and injecting into an HPLC system (Agilent 1100, Agilent Technologies Co., Ltd., Santa Clara, CA, USA) equipped with a C18 column (Agilent, USA; 4.6 mm × 250 mm, 5 µm). A mobile phase of methanol and ultrapure water (15:85), a flow rate of 1.0 mL/min, and an injection volume of 20 μL were used. Detection was carried out using a DAD detector set to a wavelength of 280 nm. The 5-HMF concentrations (mg/L) were determined using a calibration curve (*y* = 0.254*x* + 0.161, *R*^2^ = 0.9996) using OpenLab CDS 2.8 (Agilent, USA).

### 2.7. Measurement of Fat Oxidation

Two different analyses were conducted to determine the peroxide value (POV) and thiobarbituric acid reactive substances (TBARSs) in casein powder. The POV was determined following the procedure of Wang et al. [23]. To begin, a 2.0 g sample of casein powder was mixed with a 30 mL mixture of chloroform and acetic acid in a 2:3 ratio. The resulting mixture was shaken for 20 min at room temperature. Next, 0.5 mL of saturated potassium iodide (KI) solution was added to the mixture. After keeping the mixture in darkness for 5 min, 75 mL of distilled water was added, followed by the addition of 0.5 mL of starch solution (1%, *w*/*v*) as an indicator. The POV was then determined by titrating the iodine produced from the KI solution using a 0.01 M solution of standard sodium thiosulfate. The POV result is expressed in milliequivalents peroxide/kg in casein powder.

The TBARS analysis was carried out according to Wang and Wang et al. [8] with some modifications. A 2.0 g sample of casein powder was completely dissolved in an alkaline solution (pH 8.0) at 65 °C and mixed with trichloroacetic acid (5.0%, *w*/*v*) and a thiobarbituric acid solution (1.0%, *v*/*v*) in a 1:1 (*v*/*v*) ratio. The mixture was then shaken vigorously at 90 °C for 45 min. Following cooling, the mixture was centrifuged at 3000× *g* rpm for 5 min, and the absorbance was taken immediately at 532 nm using a spectrophotometer (UV2550, Shimadzu Co., Ltd., Kyoto, Japan). The TBARS is expressed as mg malondialdehyde (MDA)/kg in casein.

### 2.8. Solubility

Casein solubility was determined using the method of Liu et al. [3] with slight modifications. Briefly, 6 mL of casein solution (pH 8.0 adjusted by 1 M NaOH) was stirred at room temperature for 2 min and then centrifuged at 12,000× *g* for 20 min at 20 °C. The supernatant was used to determine casein solubility via the micro-Kjeldahl method (protein conversion coefficient × 6.38). The percentage of protein solubility was calculated as follows:

(5) $$\mathrm{Solubility}\;\left(\%\right)=\frac{\mathrm{protein}\;\mathrm{in}\;\mathrm{supernatant}}{\mathrm{total}\;\mathrm{protein}}\;\times 100$$

### 2.9. Emulsifying Activity

The emulsifying activity index (EAI) and emulsion stability index (ESI) of the casein were determined following the method of Liu et al. [3] with minor modifications. In brief, a 2 mg/mL protein solution dispersion (6 mL, final pH 8.0 adjusted by 1 M NaOH) and 2 mL of soybean oil were homogenized for 1 min at maximum velocity using an FB-110S high-speed homogenizer (Shanghai Litu Ultra High Voltage Equipment Co., Ltd., Shanghai, China). Subsequently, 50 μL of the emulsion was taken from the bottom of the tube at 0 min and 10 min after homogenization and diluted (1:100, *v*/*v*) in 0.1% (*w*/*v*) sodium dodecyl sulfate solution. The diluted solution was gently mixed by inverting the tube and the absorbance was measured at 500 nm using a spectrophotometer. EAI and ESI values were determined using Equations (6) and (7), respectively:

(6) $$\mathrm{EAI}\;\left(\frac{m^{2}}{g}\right)=\frac{2\;\times 2.303\;\times \;A_{0}\;\times \mathrm{dil}}{C\;\times \;\text{∅}\;\times \left(1-\theta \right)\;\times 10,000}$$

(7) $$\mathrm{ESI}\;\left(\min\right)=\frac{A_{0}}{A_{0}-A_{10}}\;\times 10$$

where dil, C, Ø, θ, A_0_, and A_10_ represent the dilution factor (100), initial concentration of casein (g/mL), optical path (0.01 m), fraction of oil used to form the emulsion (0.25), and the absorbance of the diluted emulsions at 0 and 10 min, respectively. All measurements were conducted in triplicate.

### 2.10. Statistical Analyses

The statistical analysis was performed using SPSS statistics software (SPSS Inc., IBM Corporation, Armonk, NY, USA, version 21.0) with ANOVA and Duncan’s multiple range test. The results were presented as the mean of three determinations ± standard deviation. MATLAB software (Version 7.0, MathWorks, Natick, MA, USA) was used to fit the model for drying experiments, and statistical significance was determined at a 5% probability level (*p* < 0.05). The correlation network analyses between pre-dehydration, drying methods, color, and casein properties were conducted using OmicShare tools.

## 3. Result and Discussion

### 3.1. Drying Kinetics

**Figure 3:**
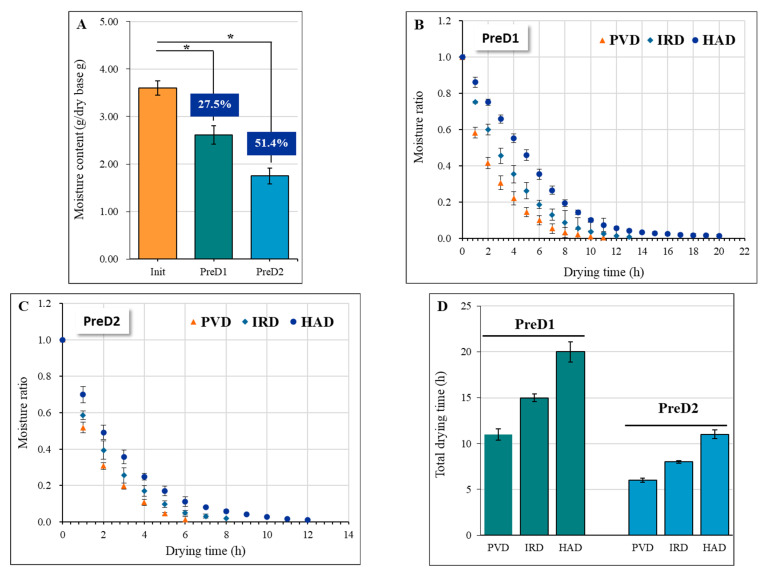
Effect of pre-dewatering intensities and drying technologies on the drying kinetics of yak milk casein. (**A**) shows the moisture content at different pressures, while (**B**,**C**) depict the drying curves with different drying methods after PreD1 and PreD2 treatment, respectively. (**D**) presents the total drying time of samples with different pretreatments and drying methods. The drying methods utilized in this study include pulsed vacuum drying (PVD), infrared drying (IRD), and hot-air drying (HAD). PreD1 and PreD2 represent the pre-dewatering of pressure 30 N and 50 N, respectively. * represents the significance at *p* < 0.05.

Physical extrusion is a promising pre-dehydration technique for yak milk casein, which can effectively remove drainable water. The moisture content of yak milk casein was significantly reduced by 27.5% and 51.4% (*p* < 0.05) after applying pressures of 30 N (PreD1) and 50 N (PreD2), respectively, under the same squeezing time of 30 min (Figure 3A). Drying was performed on the pre-dehydrated and squeezed casein, and the dehydration rate varied significantly among different drying methods. PVD exhibited the fastest drying rate, followed by IRD, while HAD was the slowest. The total drying time of PVD, IRD, and HAD was 11, 15, and 20 h under PreD1, respectively, and 6, 8, and 11 h under PreD2, respectively (Figure 3B–D). The faster drying rate of PVD can be attributed to the alternating cycle between vacuum and atmospheric pressure, which accelerates the migration rate of water from the inside of the material to the surface and thus shortens the drying time [12]. Simultaneously, the pulsed vacuum pressure is beneficial in preventing the crust-hardening effect that results from rapid water loss on the surface of the material. Similar results were observed in the drying processing of rape bee pollen [23], banana slices [24], grape [25], blueberry [12], and Goji berry [26]. Compared with HAD, the faster rate of IRD may be due to the rapid infrared radiation, which generates heat by making the molecules inside the material vibrate and rotate at high speed and thus improves the drying rate [27].

### 3.2. Mathematical Modeling

The mathematical model of one-layer drying is a useful tool for predicting water migration during material drying and is widely used in food drying research [16]. The main purpose of establishing a model is to accurately determine the endpoint of material drying and provide a theoretical basis for the development of intelligent drying technology. The suitability of different models to the drying kinetics curve depends on material properties (shape, size, texture, thickness, and maturity) and drying conditions (temperature, humidity, vacuum degree, etc.) [18]. A good fit of the mathematical model to experimental data is indicated by a higher correlation coefficient (*R*^2^), lower residual sum of squares (*RSS*), and reduced mean square of the deviation (*χ*^2^) [18]. In this study, eight mathematical models were applied to explain the thin layer drying operation of yak milk casein (Supplementary Table S1).

When the values of *R*^2^, *RSS*, and *χ*^2^ were taken into account, the Weibull and Midilli–Kucuk models were found to perform well in predicting the drying of yak milk casein, especially the Midilli–Kucuk model. The Midilli–Kucuk model was also found to be a strong fit for beetroot slices [28] and yam (*dioscorea hispida*) slices [29]. A validation test between the experimental and predicted drying rate was carried out, and the determination coefficient (*R*^2^) was higher than 0.9850, which is an acceptable level of fitting.

### 3.3. Gross Composition

The high protein content in casein makes it a valuable source of protein for various food applications. However, the reduction in protein, fat, and lactose content (0.88 g, 1.13 g, and 0.99 g/100 g dry basis, respectively) in PreD1-HAD samples may affect the nutritional value and functional properties of the final product (Table 2). The moisture content of the samples is higher after PreD1 treatment, combined with the low drying efficiency of HAD, resulting in a decrease in the overall drying rate of PreD1-HAD. The reduction in protein and lactose content may be associated with the Maillard reaction, a non-enzymatic browning reaction between reducing sugars and amino acids during prolonged drying. The decrease in fat content may be attributed to the lipid oxidation and hydrolysis reactions. These two primary adverse reactions can result in the formation of brown pigments, off-flavors, and reduced protein digestibility [30,31,32]. Therefore, it is important to optimize pre-dehydration and drying to minimize negative effects on the nutritional and functional properties of casein. During heating and drying, extended dehydration times lead to prolonged biochemical reactions, including the denaturation, oxidation, and degradation of heat-sensitive components, such as milk proteins, fats, and vitamins. These reactions ultimately have a detrimental impact on the overall quality of the end product [8,30,31]. The ash content, which represents the inorganic mineral content, is an important parameter for quality control and can affect the texture, flavor, and shelf-life of the final product [31,33]. The lack of significant differences in ash content among the different treatments indicates that the drying did not affect the mineral content of casein (Table 2).

### 3.4. Solubility and Emulsifying Activity

The solubility of caseins is a critical functional property, with high solubility being essential. It is worth noting that the samples dried using the PVD method exhibited considerably higher solubility than those obtained using IRD and HAD. The PreD2-PVD variant demonstrated the highest solubility, reaching 94.27% (Table 3). Research has shown that protein denaturation closely influences casein solubility. Long-time heat drying can lead to extensive unfolding and aggregation of caseins, resulting in decreased solubility and altered functional properties. The high solubility of PVD-dried caseins may be attributed to the retention of the native-like secondary structure of alpha-casein, beta-casein, kappa-casein, and gamma-casein compared to their HAD-dried counterparts [3]. During the drying process, caseins exhibit greater sensitivity, with samples dried using PVD and a higher pre-dewatering intensity achieving the highest EAI (6.68 m^2^·g^−1^) and ESI (12.96 min) values (Table 3). The higher solubility observed in samples dried using PVD may contribute to their superior emulsifying activity. In addition, the PVD method involves rapid drying at heat treatment, which can cause the partial denaturation of proteins. Partially denatured proteins have been proven to exhibit better emulsification properties than fully native proteins due to their ability to form more stable interfacial layers between oil and water [3,34].

### 3.5. Color Attributes

**Figure 4:**
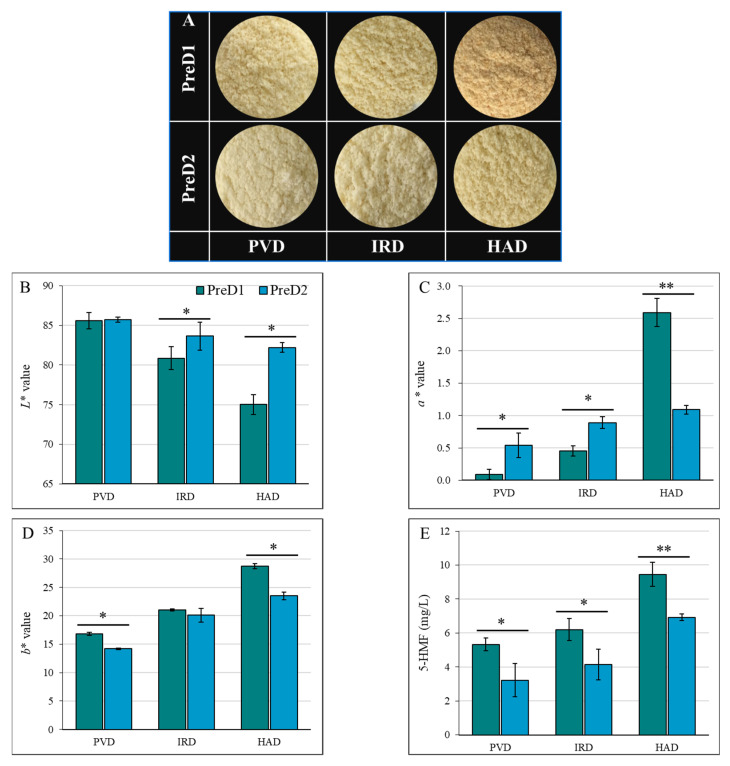
Effect of pre-dewatering intensities and drying on the color parameters of casein. PreD1 and PreD2 represent the pre-dewatering of pressure 30 N and 50 N, respectively. PVD is pulsed vacuum drying, IRD is infrared drying, and HAD is hot-air drying. (**A**) shows the picture of casein with different treatments; (**B**–**D**) show the L*, a* and b* values; (**E**) shows the 5-HMF value. * and ** represent the significance at *p* < 0.05 and *p* < 0.01, respectively.

It was shown that the L* values for PVD, IRD, and HAD significantly varied under different intensities of pre-dehydration treatment. Specifically, the L* values for PVD, IRD, and HAD were 85.57, 80.85, and 75.03, respectively, under the lower intensity of pre-dehydration treatment (PreD1). Meanwhile, the L* values for PVD, IRD, and HAD were 85.71, 83.67, and 82.19, respectively, under the higher intensity of pre-dehydration treatment (PreD2) (Figure 4A). For the same drying method, the pre-dehydration intensity had a significant impact on the color parameters a* and b*, except for the b* value of IRD. In general, the a* and b* values of the casein dried by PVD were lower, whereas those dried by HAD were higher. Under the same drying conditions, samples with higher pre-dehydration intensity (PreD2) showed higher L* and lower b* values after drying, which is mainly due to the shorter drying time under stronger pre-dehydration conditions. Shorter drying times facilitate the preservation of color by reducing the duration of color browning. The non-enzymatic browning of proteins and lactose results in the formation of brown substances. High-temperature treatment leads to pigment destruction and inactivation, such as pigment molecules in proteins like whey protein and casein [35]. Oxidation reactions cause changes in the color of certain substances, such as fatty acids and phospholipids. The presence of metal ions, such as iron and copper, participates in oxidation reactions and affects the color of dairy products [36].

The Maillard reaction is a common biochemical reaction in dairy thermal processing [35,36]. The Maillard reaction products not only affect the safety of the product but also have an important impact on the color and quality of the product. The products of the Maillard reaction are complex, among which 5-HMF serves as a characteristic reaction product and can well represent the degree of Maillard reaction [35,37]. From Figure 4E, the intensity of pre-dehydration had a significant influence on the content of 5-HMF. Lower 5-HMF content was found in the casein sample with a higher intensity of pre-dehydration, which may be attributed to the faster drying rate that reduced the occurrence of the Maillard reaction. This result of 5-HMF confirmed the changes in protein and lactose content among different PreD and dried caseins.

In summary, the color of casein is affected by various factors, including the pre-dehydration intensity, drying method, temperature, and drying retention time. The results of this study show that higher pre-dehydration intensity led to higher L* values, indicating lighter color, and resulted in lower 5-HMF content, indicating less Maillard reaction. Therefore, the optimization of the pre-dehydration and drying conditions is crucial for achieving the desired color and quality of casein.

### 3.6. Fat Oxidation

**Figure 5:**
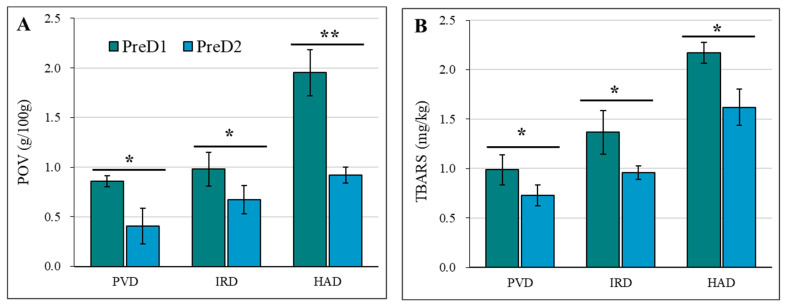
Effect of pre-dewatering intensities and drying on the fat oxidation of casein. PreD1 and PreD2 represent the pre-dewatering of pressure 30 N and 50 N, respectively. PVD is pulsed vacuum drying, IRD is infrared drying, and HAD is hot-air drying. (**A**) shows the POV value; (**B**) shows the TBARS value. * and ** represent the significance at *p* < 0.05 and *p* < 0.01, respectively.

The study investigated the effect of different pre-dewatering and drying methods on the fat oxidation of casein, as illustrated in Figure 5. The peroxide value (POV) was used as a crucial indicator to assess the extent of lipid peroxidation. The POV was measured to determine the oxidative state of the food, with a lower POV indicating a higher resistance to oxidation and a longer shelf-life [38]. The results show that a higher pre-dehydration intensity (PreD2) can result in better oxidative stability of casein during drying. This may be due to the fact that a higher pre-dehydration intensity can reduce the exposure of casein to heat treatment and oxygen during drying, which can lead to reduced fat oxidation [38,39]. All dried casein samples with PreD2 showed a lower POV compared to those with PreD1 (Figure 5A). The POVs in casein with PreD1-PVD, PreD1-IRD, PreD1-HAD, PreD2-PVD, PreD2-IRD, and PreD2-HAD treatment were 0.86, 0.98, 1.95, 0.41, 0.67, and 0.92 g/100 g, respectively.

Dairy products have a high content of unsaturated fatty acids, around 23–25%, making them prone to auto-oxidation [22]. TBARS values are a common method for assessing the amount of secondary oxidation products generated during lipid oxidation, and they have a significant impact on the sensory characteristics of dairy products [38,39]. The TBARS results are consistent with the first-order oxidation trend of fat. The pre-dehydration intensity and drying method both have significant effects on TBARS values. All dried casein samples with PreD2 showed a lower TBARS value compared to those with PreD1 (Figure 5B). The values of TBARS in casein with PreD1-PVD, PreD1-IRD, PreD1-HAD, PreD2-PVD, PreD2-IRD, and PreD2-HAD treatment were 0.16, 0.19, 0.31, 0.09, 0.14, and 0.21 mg MDA/kg, respectively (Figure 5B).

In dairy products, lipid oxidation can be accelerated by various factors, such as high temperature, light, metal ions, and enzymes [23,38,40]. During the processing of dairy products, such as pre-dehydration and drying, the exposure of milk fat to heat treatment, oxygen, and long-term drying processing can lead to increased lipid oxidation. Therefore, it is important to optimize the processing conditions to minimize the exposure of milk fat to these factors and ensure the quality and shelf-life of dairy products. In summary, lipid oxidation is a major concern in the processing and storage of dairy products. It can lead to the deterioration of product quality and safety; therefore, it is important to optimize processing conditions and use appropriate antioxidants to minimize lipid oxidation and ensure the quality and shelf-life of dairy products.

### 3.7. Correlation Analysis

**Figure 6:**
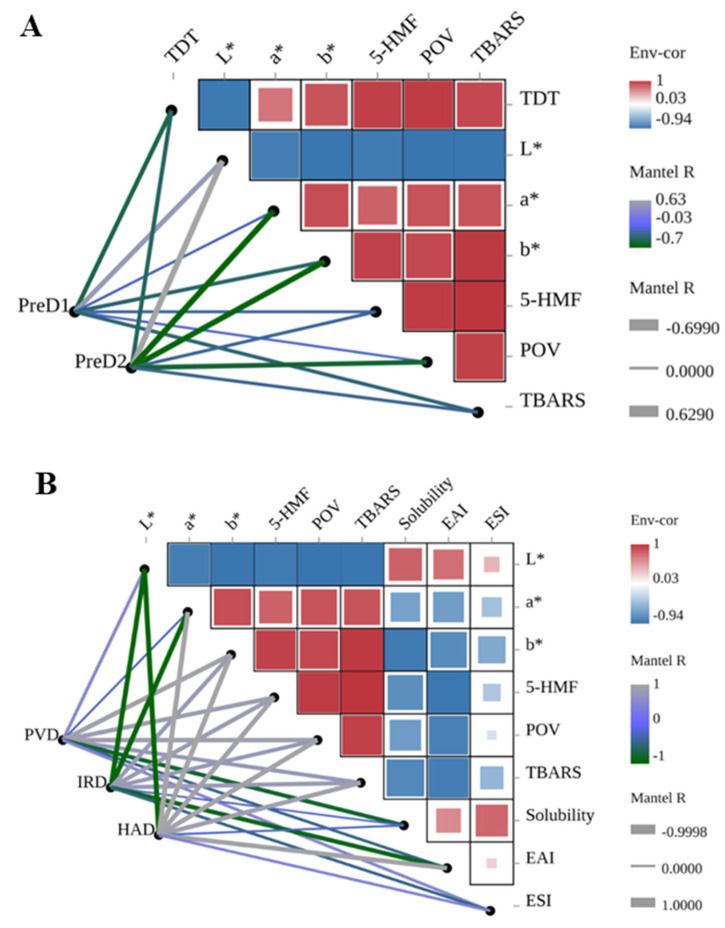

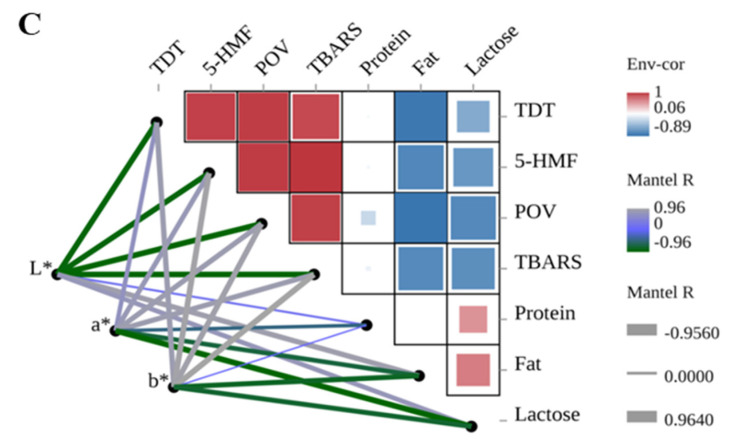
Correlation analysis of pre-dewatering intensities, drying methods, color, and casein properties. TDT is total drying time. PreD1 and PreD2 represent the pre-dewatering of pressure 30 N and 50 N, respectively. PVD is pulsed vacuum drying, IRD is infrared drying, and HAD is hot-air drying. (**A**) shows the correlation between pre-dewatering intensities and casein properties; (**B**) shows the correlation between drying methods and casein properties; (**C**) shows the correlation between color and casein properties.

To further explore the inherent relationship among different indicators, Spearman correlation analysis between pre-dehydration, drying, color, and various indicators was conducted, as shown in Figure 6. The thickness of the lines represents the correlation degree, and the color gradient from gray to green represents positive and negative correlations. It can be observed that the pre-dehydration intensity is negatively correlated with all the analyzed indicators except for the L* value (Figure 6A). This suggests that lower pre-dehydration intensity can lead to changes in the color, 5-HMF content, lipid oxidation, and emulsion stability of casein following subsequent drying.

The drying methods have a significant impact on the color, 5-HMF content, lipid oxidation, and emulsion stability of casein (Figure 6B). In terms of PVD, IRD, and HAD, the HAD method was found to have a significant negative correlation with the L* value (*r* = −0.999, *p* = 0.0005) and a significant positive correlation with the a* value, b* value, 5-HMF, POV, TBARS, and EAI (*r* > 0.913, *p* < 0.01). This indicates that HAD has a significant effect on the color, Maillard reaction, lipid oxidation, and emulsion stability of casein.

The drying time, 5-HMF content, lipid oxidation, composition (protein, fat, and lactose), and color of casein are also correlated (Figure 6C). The a* value was found to be significantly positively correlated with lipid oxidation (*r* > 0.840, *p* < 0.03) and negatively correlated with lactose (*r* = −0.956, *p* = 0.003), while the b* value was positively correlated with drying time, 5-HMF content, and lipid oxidation (*r* > 0.839, *p* < 0.03) and negatively correlated with fat content (*r* = −0.813, *p* = 0.049).

Overall, pre-dehydration intensity is positively correlated with the downstream drying rate, and the quality of casein is mainly influenced by the drying method and pre-dehydration. Therefore, it is important to carefully select the pre-dehydration intensity and drying method to minimize the changes in the color, Maillard reaction, lipid oxidation, and emulsion stability of casein and ensure its quality and shelf-life.

## 4. Conclusions

The study aimed to evaluate the effects of pre-dehydration and various drying methods on the drying kinetics and quality attributes of yak milk casein. The results show that increasing extrusion pre-dehydration pressure to 50 N significantly improved the drying rate, resulting in shorter drying times across all drying methods. The Midilli–Kucuk mathematical model effectively described the drying of yak milk casein. Hot-air drying with a long drying time led to higher fat oxidation, lower solubility, and emulsifying activity compared to the other methods. Additionally, color degradation was more pronounced in hot-air-dried products due to the Maillard reaction and fat oxidation.

In summary, the integration of extrusion pre-dehydration and pulsed vacuum drying technology has been successful in enhancing the drying rate of casein while preserving the quality of the final product. The extrusion pre-dehydration pressure at 50 N was more effective than treatment at 30 N, and a pulsed vacuum drying temperature of 40 °C is recommended. This approach is highly promising for its potential use in industrial settings, as well as in the production of high-quality artisanal casein in small-scale rural households.

## Figures and Tables

**Table 1:** The thin layer drying models utilized in the study.

No.	Model Name	Model	References
1	Page	$MR=\exp\left(-{kt}^{n}\right)$	[16]
2	Newton	MR=exp(−kt)	[17]
3	Henderson and Pabis	MR=aexp(−kt)	[18]
4	Logarithmic	MR=aexp(−kt) + c	[19]
5	Verma	$MR=a\exp\left(-\mathrm{kt}\right)+(1\;-\;a)\exp\left(-\mathrm{gt}\right)$	[16]
6	Two-term exponential	$MR=a\exp\left(-\mathrm{kt}\right)+(1\;-\;a)\exp\left(-\mathrm{kat}\right)$	[20]
7	Weibull	$MR=\exp\left[-{\left(t/\alpha \right)}^{\beta }\right]$	[21]
8	Midilli–Kucuk	$MR=a\exp\left(-kt^{n}\right)+\mathrm{bt}$	[19]

Here, *t* is the drying time (hr); *k* = drying constant (hr^−1^); *n*, *a*, *g*, *b*, *α*; and *β* is the drying coefficients.

**Table 2:** Gross composition of yak milk casein with different pre-dewatering intensities and drying methods.

Pre-Dewatering	Drying Method	Gross Composition (g/100 g Dry Basis)			
		Protein	Fat	Lactose	Ash
Control		72.03 ± 1.71 ^a^	6.11 ± 0.29 ^a^	5.68 ± 0.44 ^a^	5.35 ± 0.99
PreD1	PVD	71.42 ± 2.56 ^bc^	6.32 ± 0.23 ^bc^	5.40 ± 0.19 ^a^	5.27 ± 0.15
	IRD	71.63 ± 1.09 ^bc^	5.16 ± 0.62 ^bc^	5.51 ± 0.32 ^a^	5.31 ± 0.17
	HAD	71.15 ± 1.20 ^c^	4.80 ± 0.31 ^c^	4.69 ± 0.70 ^b^	5.27 ± 0.11
PreD2	PVD	71.14 ± 2.17 ^c^	5.62 ± 0.20 ^ab^	5.34 ± 0.21 ^ab^	5.23 ± 0.19
	IRD	71.41 ± 2.61 ^bc^	5.19 ± 1.03 ^bc^	5.13 ± 0.09 ^ab^	5.25 ± 0.28
	HAD	71.54 ± 1.46 ^bc^	5.43 ± 0.33 ^bc^	5.17 ± 0.26 ^ab^	5.18 ± 0.23

PreD1 and PreD2 represent the pre-dewatering of pressure 30 N and 50 N, respectively. PVD is pulsed vacuum drying, IRD is infrared drying, and HAD is hot-air drying. ^a,b,c^ Means in the same column with different superscripts differ significantly (*p* < 0.05).

**Table 3:** Solubility and emulsifying activity of the yak milk casein with different pre-dewatering intensities and drying methods.

Pre-Dewatering	Drying Method	Solubility (%)	EAI (m^2^·g^−1^)	ESI (min)
PreD1	PVD	91.27 ± 1.01 ^b^	6.14 ± 0.09 ^bcd^	13.45 ± 0.75 ^a^
	IRD	85.70 ± 1.82 ^c^	6.40 ± 0.43 ^abc^	12.42 ± 1.34 ^ab^
	HAD	83.60 ± 1.95 ^c^	5.68 ± 0.46 ^d^	12.38 ± 0.62 ^ab^
PreD2	PVD	94.27 ± 1.66 ^a^	6.68 ± 0.15 ^a^	12.96 ± 0.72 ^ab^
	IRD	86.33 ± 2.10 ^c^	6.64 ± 0.19 ^ab^	12.20 ± 0.99 ^ab^
	HAD	84.27 ± 1.26 ^c^	5.96 ± 0.08 ^cd^	11.37 ± 1.09 ^b^

EAI means emulsifying activity index, ESI means emulsion stability index. PreD1 and PreD2 represent the pre-dewatering of pressure 30 N and 50 N, respectively. PVD is pulsed vacuum drying, IRD is infrared drying, and HAD is hot-air drying. Different letters ^a–d^ in the same column are significantly different (*p* < 0.05).

## Data Availability

The original contributions presented in the study are included in the article and Supplementary Materials, further inquiries can be directed to the corresponding author.

## Supplementary Materials

The following supporting information can be downloaded at: https://www.mdpi.com/article/10.3390/foods13071062/s1. Supplementary Table S1. Mechanistic model parameters and the results of the statistical computations for drying of yak milk casein.
