# Supplementary material for: Impact of the Pre-Dehydration and Drying Methods on the Mass Transfer and Quality Attributes of Yak Milk Casein
Source: Foods. 2024 Mar 29;13(7):1062. doi: 10.3390/foods13071062 (PMC11012072; doi:10.3390/foods13071062)
Supplement: Supplementary file 1 [file foods-13-01062-s001.zip › foods-2902947-supplementary.pdf]

Supplementary Table S1. Mechanistic model parameters and the results of the statistical computations for drying of yak milk casein.

Mathematical model	Pre-dewatering	Drying method	Parameters of the model	R^2	RSS (×10^{-3})	χ^2 (×10^{-4})
Page	PreD1	PVD	k=0.2409, n=1.0922	0.9980	2.7912	1.9912
		IRD	k=0.4902, n=0.8631	0.9962	3.7501	3.7478
		HAD	k=0.0998, n=1.3287	0.9972	5.5835	2.9353
	PreD2	PVD	k=0.5043, n=0.9364	0.9984	1.3810	1.9752
		IRD	k=0.6351, n=0.9165	0.9983	1.4204	2.8499
		HAD	k=0.3492, n=1.0072	0.9998	0.2414	0.2185
Newton	PreD1	PVD	k=0.2773	0.9961	5.4006	3.6025
		IRD	k=0.4181	0.9914	8.5212	7.7490
		HAD	k=0.1851	0.9795	4.1051	20.5000
	PreD2	PVD	k=0.4730	0.9975	2.1300	2.6589
		IRD	k=0.5946	0.9968	2.3727	3.9490
		HAD	k=0.3524	0.9998	0.2533	0.2111
Henderson and Pabis	PreD1	PVD	k=0.2807, a=1.0130	0.9963	5.1417	3.6740
		IRD	k=0.4043, a=0.9667	0.9928	7.1703	7.1696
		HAD	k=0.1964, a=1.0670	0.9839	32.2100	17.0142
	PreD2	PVD	k=0.4672, a=0.9871	0.9977	1.9301	2.7593
		IRD	k=0.5887, a=0.9893	0.9969	2.2415	4.4891
		HAD	k=0.3524, a=1.0001	0.9998	0.2533	0.2303
Logarithmic	PreD1	PVD	k=0.2540, a=1.0336, c=-0.0340	0.9988	1.7017	1.3053
		IRD	k=0.4103, a=0.9642, c=0.0043	0.9928	7.1204	7.9115
		HAD	k=0.1681, a=1.1044, c=-0.0597	0.9897	20.7011	11.5720
	PreD2	PVD	k=0.4665, a=0.9874, c=-0.0005	0.9977	1.9310	3.2185
		IRD	k=0.5948, a=0.9866, c=0.0035	0.9969	2.2335	5.5812
		HAD	k=0.3479, a=1.0026 c=0.3479	0.9998	0.2073	0.2073
Verma	PreD1	PVD	k=0.3967, a=83.4213, g=0.3987	0.9985	2.1524	1.6552
		IRD	k=0.3552, a=0.8456, g=37.2020	0.9982	1.76434	1.9564
		HAD	k=0.3372, a=118.6083, g=0.3394	0.9971	5.9020	3.2803
	PreD2	PVD	k=0.4733, a=4.9598, g=0.4734	0.9975	2.1300	3.5452
		IRD	k=0.5359, a=0.8939,	0.9986	1.0516	2.6325

			g=6573.5263			
		HAD	k=0.3993, a=19.1320, g=0.4021	0.9998	0.2184	0.2184
Two term exponential	PreD1	PVD	k=0.3369, a=1.5547	0.9983	2.3922	1.7082
		IRD	k=0.4130, a=0.9987	0.9913	8.6101	8.6088
		HAD	k=0.2649, a=1.8614	0.9964	7.2710	3.8269
	PreD2	PVD	k=0.5860, a=0.6041	0.9978	1.9035	2.7086
		IRD	k=0.8600, a=0.5047	0.9975	1.8616	3.7209
		HAD	k=0.3724, a=1.2594	0.9998	0.2207	0.2006
Weibull	PreD1	PVD	α=3.6805, β=1.0925	0.9980	2.7910	1.9911
		IRD	α=2.2842, β=0.8634	0.9962	3.7516	3.7477
		HAD	α=5.6674, β=1.3294	0.9972	5.5823	2.9352
	PreD2	PVD	α=2.0775, β=0.937	0.9984	1.3858	1.9752
		IRD	α=1.6409, β=0.9164	0.9981	1.4200	2.8499
		HAD	α=2.8424, β=1.0072,	0.9998	0.2404	0.2185
Midilli-Kucuk	PreD1	PVD	a=0.9911, b=-0.0114, k=0.2430, n=1.0596	0.9988	1.6635	1.3868
		IRD	a=0.9980, b=-0.0046, k=0.5081, n=0.7687	0.9987	1.2675	1.5719
		HAD	a=0.9692, b=3.9402×10^{-4} k=0.0827, n=1.4175	0.9978	4.3545	2.5560
	PreD2	PVD	a=0.9989, b=-0.0047, k=0.5125, n=0.8605	0.9994	0.5201	1.0401
		IRD	a=1.0000, b=-0.0106, k=0.6377, n=0.7837	0.9998	0.1289	0.4295
		HAD	a=0.9988, b=-3.3475×10^{-4}, k=0.3500, n=0.9995	0.9998	0.2122	0.2358

PreD1 and PreD2 represents the pre-dewatering of pressure 30 N and 50 N, respectively. PVD is pulsed vacuum drying, IRD is infrared drying, and HAD is hot-air drying.
